# Targeted Co-delivery of Tumor Antigen and α-Galactosylceramide to CD141^+^ Dendritic Cells Induces a Potent Tumor Antigen-Specific Human CD8^+^ T Cell Response in Human Immune System Mice

**DOI:** 10.3389/fimmu.2020.02043

**Published:** 2020-08-18

**Authors:** Jing Huang, Jing Zhou, Reem Ghinnagow, Toshiyuki Seki, Sho Iketani, Daphnée Soulard, Patrick Paczkowski, Yukiko Tsuji, Sean MacKay, Luis Javier Cruz, François Trottein, Moriya Tsuji

**Affiliations:** ^1^Aaron Diamond AIDS Research Center, The Rockefeller University, New York, NY, United States; ^2^Department of Medicine, Columbia University Irving Medical Center, New York, NY, United States; ^3^IsoPlexis, Branford, CT, United States; ^4^Centre d’Infection et d’Immunité de Lille, Inserm U1019, CNRS UMR 8204, CHU Lille, Institut Pasteur de Lille, University of Lille, Lille, France; ^5^Department of Obstetrics and Gynecology, The Jikei University School of Medicine, Tokyo, Japan; ^6^Department of Microbiology and Immunology, Columbia University Irving Medical Center, New York, NY, United States; ^7^Translational Nanobiomaterials and Imaging, Department of Radiology, Leiden University Medical Center, Leiden, Netherlands

**Keywords:** nanovaccine, targeting, human CD141^+^ DCs, *i*NKT cells, α-galactosylceramide, CD8^+^ T cells, human immune system mice, melanoma

## Abstract

Active co-delivery of tumor antigens (Ag) and α-galactosylceramide (α-GalCer), a potent agonist for invariant Natural Killer T (*i*NKT) cells, to cross-priming CD8α^+^ dendritic cells (DCs) was previously shown to promote strong anti-tumor responses in mice. Here, we designed a nanoparticle-based vaccine able to target human CD141^+^ (BDCA3^+^) DCs - the equivalent of murine CD8α^+^ DCs – and deliver both tumor Ag (Melan A) and α-GalCer. This nanovaccine was inoculated into humanized mice that mimic the human immune system (HIS) and possess functional *i*NKT cells and CD8^+^ T cells, called HIS-CD8/NKT mice. We found that multiple immunizations of HIS-CD8/NKT mice with the nanovaccine resulted in the activation and/or expansion of human CD141^+^ DCs and *i*NKT cells and ultimately elicited a potent Melan-A-specific CD8^+^ T cell response, as determined by tetramer staining and ELISpot assay. Single-cell proteomics further detailed the highly polyfunctional CD8^+^ T cells induced by the nanovaccine and revealed their predictive potential for vaccine potency. This finding demonstrates for the first time the unique ability of human *i*NKT cells to license cross-priming DCs *in vivo* and adds a new dimension to the current strategy of cancer vaccine development.

## Introduction

The development of cancer vaccines has been a challenging – but promising – task to date. The induction of protective cytotoxic CD8^+^ T (CTL) cells against tumors has been the main objective of these vaccines (1–5). To achieve this goal, many attempts have been undertaken, including the optimization of the vaccine formulation and vaccine delivery, as well as the inclusion of adjuvants (4–9). One well-studied approach is the use of poly(lactic-co-glycolic) acid (PLGA)-based nanoparticles (NP) as a vaccine delivery system. These NPs have several desired properties, including high antigen density, incorporation of different classes of molecules including proteins and lipids, ability to reach the MHC-class I pathway after uptake by dendritic cells (DCs), and slow extended release of their payload (10, 11). PLGA-based NP vaccines can also be targeted to specific cell types; for example, to CD141^+^ (BDCA3^+^) cross-priming DCs by using anti-CLEC9A antibodies (Abs) (12). In this regard, it was previously shown that the presentation of tumor antigens (Ag) by cross-priming DCs can effectively induce a potent anti-tumor CTL response (1, 3, 4). Given this potential, some tumor vaccines have been designed for targeting cross-priming DCs (13–16), one of which is now in clinical development (17). A further benefit of these NP vaccines, also known as nanovaccines, is that they can co-encapsulate tumor Ag and immune modulators (adjuvants), such as a Toll-like receptor (TLR) agonist for the purpose of enhancing the tumor-specific CD8^+^ T cell response (18, 19). Alpha-galactosylceramide (α-GalCer), a strong agonist of invariant natural killer T (*i*NKT) cell cells, is known to act as a potent adjuvant (20–24). In response to α-GalCer, *i*NKT cells swiftly produce immunostimulatory cytokines, such as IFN-γ, which in turn leads to DC maturation and activation and ultimately triggers the downstream activation of effector cells, such as NK cells and T lymphocytes (20–24). This α-GalCer has also been co-encapsulated with a tumor antigen (12, 25).

We have previously designed a PLGA-based NP vaccine by encapsulating a tumor Ag and α-GalCer and decorating the NPs with Abs against human CLEC9A to target cross-priming CD141^+^ DCs known to be capable of inducing potent CTL responses (13, 16, 26). Using nanovaccines co-encapsulating tumor self-Ag (Trp2 and gp120) and α-GalCer, and decorated with Abs against Clec9a (mouse counterpart), we have recently shown that these nanovaccines induce a potent CTL response that protects in prophylactic and therapeutic settings against the development of aggressive tumors (melanoma) (12). We have also shown that the NP vaccine containing the Melan A Ag and α-GalCer and coated with anti-CLEC9A Abs strongly induced the expansion of tumor Ag-specific CD8^+^ T cells from human PBMCs *in vitro* (12). Although these studies have clearly revealed that the adjuvant functions of *i*NKT cells can be exploited by the simultaneous co-delivery of both α-GalCer and Ag into the same DCs, the *i*NKT cell helper functions have yet to be tested in a human setting *in vivo*. Our current objective was to confirm these promising data *in vivo* by using a humanized mouse model. To this end, we took advantage of a novel model of humanized mice, termed human immune system (HIS) mice, which possess functional human *i*NKT cells and CD8^+^ T cells, called HIS-CD8/NKT mice (27). These HIS-CD8/NKT mice were generated by the transduction of β2-microglobulin (β2m)-deficient NOD/SCID*-IL2r*γ*^null^* (NSG) mice with adeno-associated virus serotype 9 (AAV9) expressing genes that encode HLA-A*0201 linked to human β2m, human CD1d linked to human β2m, and also human hematopoietic cytokines (human GM-CSF, IL-3, and IL-15). Then, these human genes-transduced NSG mice were engrafted with HLA-A*0201-positive human hematopoietic stem cells as a source of various human immune-competent cells (28). It is important to note that we recently were able to exhibit the functionality of human CD141^+^ DCs in our HIS mice, validating their utility for this study (29). Here, using a nanovaccine loaded with the tumor Ag Melan A and α-GalCer and decorated with anti-CLEC9A Abs, we aimed to analyze the immune response in HIS-CD8/NKT mice. Our data confirm the exquisite ability of the vaccine to expand/activate CD141^+^ DCs and *i*NKT cells and ultimately to generate a potent Melan-A-specific CD8 + T cell response.

## Materials and Methods

### Ethics Statement

All animal experiments were conducted in strict accordance with the Policy on Humane Care and Use of Laboratory Animals of the United States Public Health Service. The protocol was approved by the Institutional Animal Care and Use Committee at the Rockefeller University (Assurance # A3081-01). CO_2_ was used for euthanasia, and all efforts were made to minimize suffering. Human fetal liver samples were obtained via a non-profit partner (Advanced Bioscience Resources, Alameda, CA, United States); the samples were devoid of any information that would identify the subjects from whom they were derived. Therefore, IRB approval was not required for their use, as previously described (27–29).

### Mice

NOD.Cg-B2m^tm1Unc^ Prkdc^scid^ Il2rg^tm1Wjl^/SzJ (NSG-B2m) triple mutant mice, which combined the features of severe combined immune deficiency mutation with IL-2 receptor γ chain and β2-microglobulin (β2m) deficiencies, were purchased from The Jackson Laboratories. Mice were maintained under specific pathogen-free conditions in the animal facilities at the Comparative Bioscience Center of The Rockefeller University, as previously described (27–29).

### Generation of HIS-CD8/NKT Mice

**FIGURE 1:**
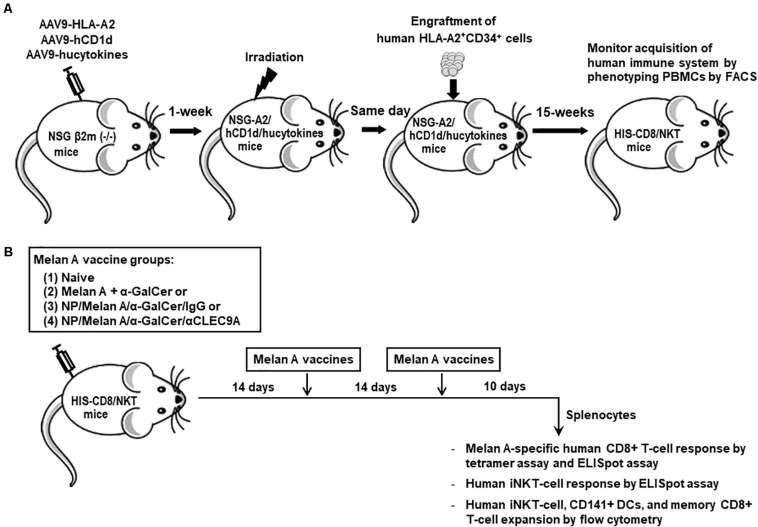
Establishment of HIS-CD8/NKT mice and schematic representation of the immunization protocol. **(A)** NSG-B2m mice were first transduced with HLA-A2, human CD1d, and selected human cytokines, followed by sub-lethal irradiation and engraftment of HLA-A2^+^ HSCs after 1 week. Fifteen weeks after the HSC engraftment, the status of HIS reconstitution was determined by measuring the percentages of certain human lymphocyte subpopulations present in PBMCs by flow cytometry. **(B)** After the establishment of HIS-CD8/NKT mice, they were immunized with NP-based Melan-A vaccines three times with 2-week intervals and 10 days after the last boost, splenocytes were isolated and various immunoassays were performed.

Three-week old NSG-B2m mice, which lack endogenous mouse CD1d, were given intravenous (i.v.) injection of adeno-associated virus serotype 9 (AAV9) which encodes for human IL-3, IL-15, and GM-CSF, as well as peri-thoracic injection of AAV9 which encodes for HLA-A*0201 (28) and human CD1d (27). One to two weeks after the transduction of selected human genes by AAV9 vectors, mice were exposed to 150-Gy whole-body sublethal irradiation for myeloablation. A few hours later, each transduced, irradiated mouse was engrafted i.v. with 1 × 10^5^ HLA-A*0201^+^ matched CD34^+^ human HSCs which were isolated from fetal liver samples, as previously described (27–29). The entire procedure of generating HIS-CD8/NKT mice is illustrated in Figure 1A.

### Construction of PLGA-Based NP Vaccines That Co-deliver Melan A and α-GalCer With or Without Anti-CLEC9A Antibody Attachment

PLGA-based NP coated with lipid-polyethylene glycol and carrying Ab were generated using the copolymer PLGA essentially as recently described (12). Briefly, 20 mg of endotoxin-free 18-mer Melan A peptide, which contains the HLA-A*0201-restricted CD8^+^ T-cell epitope (ELAGIGILTV), and 50 μg of α-GalCer were co-encapsulated to 100 mg of PLGA. Anti-human CLEC9A Ab or an isotype control was attached to the lipid-polyethylene glycol layer. The PLGA-based NPs were then characterized by dynamic light scattering and zeta potential to assess quality. Incorporation of Melan A peptide in NPs was quantified by Coomassie dye protein assay (Thermo Fisher Scientific, United States) or by reversed-phase high-performance liquid chromatography. The presence of Ab on the particle surface was quantified by Coomassie dye protein assay. The constructed NP/MelanA/α-GalCer/IgG and NP/MelanA/α-GalCer/anti-CLEC9A vaccines were designed to be analogous to those which were previously used to investigate the *in vitro* activation of the α-GalCer-reactive human iNKT-cell response, as well as Melan-A-specific human CD8^+^ T-cell proliferation of the PBMCs collected from HLA-A2^+^ healthy donors and melanoma patients (12).

### Immunization Regimens

The immunization regimens were selected based on our previously published studies (12), in which a free α-GalCer/tumor peptides complex, NP/α-GalCer/tumor peptides/IgG, and NP/α-GalCer/tumor peptides/anti-Clec9a were immunized 3 times and tumor-specific mouse CD8+ T-cell response, as well as α-GalCer-reactive mouse iNKT-cell response, were measured (12). Regimens were administered by the intramuscular route, because this route is one of the three parenteral routes (subcutaneous, intradermal, and intramuscular) approved by the U.S. Food and Drug Administration (FDA) and European Medicines Agency (EMA) for licensed PLGA to be used in humans (11). Therefore, in order to test the immunogenicity of the NP vaccine which co-delivers Melan A and α-GalCer and is decorated by anti-CLEC9A Ab (NP/Melan A/α-GalCer/anti-CLEC9A), a group of HIS-CD8/NKT mice were immunized three times i.m. with the vaccine with 2-week intervals (Figure 1B). An NP vaccine that co-delivers Melan A peptide and α-GalCer and is coated by an isotype IgG (12), as well as the mixture of soluble forms of Melan A peptide and α-GalCer, were immunized into other groups of HIS-CD8/NKT mice as controls. Ten days after the last immunization, splenocytes were isolated from the spleens of immunized, as well as naïve HIS-CD8/NKT mice, for analysis.

### A Flow Cytometric Analysis to Determine the Phenotypes of Human Lymphocyte Subsets in the Spleen of Immunized, as Well as Naïve HIS-CD8/NKT Mice

Splenocytes isolated from immunized and naïve HIS-CD8/NKT mice were blocked for 5 min on ice using normal mouse sera supplemented with anti-CD16/CD32 (clone 93, BioLegend) (27–29). Cells were washed once and stained for 40 min on ice in the dark with the following antibodies: Pacific Blue anti-human CD45 (clone HI30, BioLegend, San Diego, CA, United States), Pacific Orange anti-mouse CD45 (clone 30-F11, Life Technologies, Carlsbad, CA, United States), phycoerythrin (PE)-TexasRed antihuman CD3 (clone UCHT1, Life Technologies), allophycocyanin (APC)-Cy7 anti-human CD4 (clone RPA-T4, BioLegend), fluorescein isothiocyanate (FITC) anti-human CD8 (clone HIT8a, BioLegend), peridinin chlorophyll protein complex (PerCp)-Cy5.5 anti-human TCR Vα24/Jα18 (clone 6B11, BioLegend), Alexa Fluor 647 anti-human CD161 (clone HP-3G10, BioLegend), PE-Cy7 anti-human CD19 (clone HIB19, BioLegend), PE anti-CD11c (clone 3.9, BioLegend), and PerCp-Cy5.5 anti-human CD14 (clone M5E2, BioLegend). After staining, cells were washed twice with PBS containing 2% FBS, fixed with 1% paraformaldehyde, and analyzed using a BD LSR II Flow Cytometer (BD Biosciences, Franklin Lakes, NJ, United States).

### Tetramer Staining to Determine Melan-A-Specific HLA-A*0201-Restricted CD8^+^ T-Cell Response

Splenocytes were isolated from immunized and naïve HIS-CD8/NKT mice and incubated with Melan A peptide-loaded HLA-A*0201 tetramer, which was kindly supplied by the NIH Tetramer Core Facility. We also incubated the cells with the following antibodies: Pacific Blue anti-human CD45 (clone HI30), Pacific Orange anti-mouse CD45 (clone 30-F11), PE-Texas Red anti-human CD3 (clone UCHT1), APC-Cy7 anti-human CD4 (clone RPA-T4), FITC anti-human CD8 (clone HIT8a), and PE-Cy7 anti-human CD19 (clone HIB19). Finally, the percentage of Melan-A-specific human CD8^+^ T cells was determined using a BD LSR II Flow Cytometer.

### An ELISpot Assay to Determine Melan-A-Specific Human CD8^+^ T-Cell Response and Human *i*NKT-Cell Response

After isolation of splenocytes, the relative numbers of Melan-A-specific IFN-γ-secreting human CD8^+^ T cells or α-GalCer-reactive human *i*NKT cells among 1 × 10^6^ splenocytes obtained from immunized and naïve HIS-CD8/NKT mice were determined using ELISpot assays (27). Briefly, 1 × 10^6^ splenocytes were placed on each well of the 96-well ELISpot plate pre-coated with captured anti-human IFN-γ antibody and incubated for 24 h in the presence of a synthetic peptide corresponding to the HLA-A*0201-restricted CD8^+^ T-cell epitope of Melan A antigen (ELAGIGILTV) (12) for detecting Melan-A-specific CD8^+^ T cells, or α-GalCer (27) for detecting human iNKT cells. Then, the ELISpot plate was incubated with biotinylated anti-human IFN-γ antibody, followed by incubation with avidin-conjugated with horseradish peroxidase. Finally, the spots were developed after adding ELISpot substrate (BioLegend), as described (27, 28).

### Thirty-Two-Plex Single-Cell Proteomics Detection Platform to Profile Human CD8^+^ T-Cell Polyfunctionality

Splenic human CD8^+^ T cells were isolated from immunized and naïve HIS-CD8/NKT mice by anti-human CD8 microbeads (Miltenyi) and stimulated with plate-bound anti-human CD3 (10 μg/mL, clone OKT3) and soluble anti-human CD28 (5 μg/mL, clone CD28.2) at 37°C, 5% CO_2_ for 24 h. After stimulation, cells were stained with PE-conjugated anti-human CD8, rinsed and resuspended with fresh complete culture media and loaded on a single-cell IsoCode containing ∼12000 cellular microchambers, each of which was pre-patterned with a complete copy of the 32-plex antibody panel (30–33). After an additional 16-h-on-chip-incubation at 37°C, 5% CO_2_, proteins secreted from approximately 1000 single cells were captured and analyzed by the 32-plex ELISA array categorized into different functional groups: Effector: Granzyme B, TNF-α, IFN-γ, MIP1-α, Perforin, TNF-β; Stimulatory: GM-CSF, IL-2, IL-5, IL-7, IL-8, IL-9, IL-12, IL-15, IL-21; Chemoattractive: CCL11, IP-10, MIP-1β, RANTES; Regulatory: IL-4, IL-10, IL-13, IL-22, sCD137, sCD40L, TGF-β1; Inflammatory: IL-6, IL-17A, IL-17F, MCP-1, MCP-4, IL-1β. Protein signals from zero-cell microchambers were used to assess cytokine-specific background. Signals with a signal-to-noise ratio (SNR) of at least 2 (relative to the background threshold) and from at least 5 single cells or 2% of all single cells (whichever quantity was larger) were considered as significantly secreted. The polyfunctional profile of individual cells defined as co-secreted 2 + proteins per cell was evaluated by the IsoSpeak software package. The polyfunctional strength index (PSI) was calculated in a similar manner as previously described (34), defined as the percentage of polyfunctional cells, multiplied by mean fluorescence intensity (MFI) of the proteins secreted by those cells. The 3D t-SNE visualization of all single cells was analyzed in the IsoSpeak software by using the following hyperparameters: theta: 0.5; perplexity: 50; maximum iterations: 1000; and seed: 123.

### Statistical Analysis

All statistical analyses were performed using GraphPad Prism (ver. 7) (GraphPad Software, Inc). For all studies, unpaired *t*-test or Mann-Whitney *U* Test was used to determine the differences between two groups. For statistical differences across the four groups, an ANOVA test followed by Tukey’s HSD test were used.

## Results

### NPs Incorporating Self-Tumor Ag and α-GalCer Induce a Potent Melan-A-Specific Human CD8^+^ T-Cell Response in HIS-CD8/NKT Mice

HIS-CD8/NKT mice were generated as illustrated in Figure 1A, and then divided into four groups for immunization by the following: (1) Naïve untreated mice, (2) a mixture of soluble forms of Melan A peptide and α-GalCer, (3) NP vaccine which co-delivers Melan A peptide and α-GalCer and is coated by an isotype IgG (NP/Melan A/α-GalCer/IgG), (4) NP vaccine which co-delivers Melan A and α-GalCer and is decorated by anti-CLEC9A Ab (NP/Melan A/α-GalCer/anti-CLEC9A). Immunizations were conducted three times intramuscularly (i.m.), 2 weeks apart (Figure 1B). Of note, the anti-CLEC9A Ab used in this study reacts with only human DCs (12) and does not cross-react with mouse DCs (Supplementary Figure S1).

**FIGURE 2:**
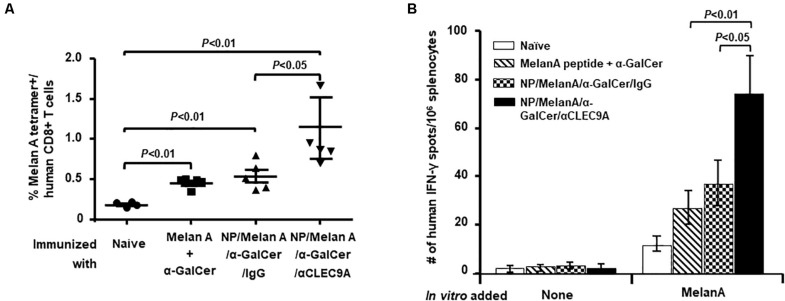
Level of Melan-A-specific, HLA-A2-restricted human CD8^+^ T-cell response induced by the NP vaccines. **(A)** The percentage of Melan-A-specific T cells among total human CD8^+^ T cells in the spleen of all groups of mice was determined by flow cytometry using the Melan A tetramer. **(B)** The relative number of Melan-A-specific, HLA-A2-restricted human CD8^+^ T cells that secrete IFN-γ among splenocytes from all groups of mice was determined by an ELISpot assay. Results are displayed as the mean value and standard error (*n* = 5). Statistical analyses were performed using ANOVA followed by Dunnett’s test and differences were considered if *P* < 0.05.

Ten days after the last administration, the expansion of the Melan A-specific human CD8^+^ T-cell response was evaluated. To this end, the percentage of human CD8^+^ T cells that were positive for Melan A peptide (ELAGIGILTV)-loaded HLA-A*0201 tetramer was assessed by flow cytometry (Figure 2A). Compared to controls, the NP vaccine having both the Melan A peptide and α-GalCer and coated with anti-CLEC9A Ab (NP/Melan A/α-GalCer/anti-CLEC9A) induced a higher level of the Melan A-specific, HLA-A*0201-restricted human CD8^+^ T-cell response (Figure 2A and Supplementary Figure S2). Although HIS-CD8/NKT mice immunized with the control NP vaccine (NP/Melan A/α-GalCer/IgG) or with a mixture of soluble Melan A and α-GalCer displayed a significantly higher percentage of Melan A-specific, HLA-A*0201-restricted human CD8^+^ T-cell response compared to naïve HIS-CD8/NKT mice, the response was significantly lower than that induced by NP/Melan A/α-GalCer/anti-CLEC9A.

We also conducted an ELISpot assay to determine the relative number of IFN-γ-secreting human CD8^+^ T cells in response to Melan-A peptide *ex vivo* (Figure 2B). We observed that HIS-CD8/NKT mice immunized with NP/Melan A/α-GalCer/anti-CLEC9A had a higher number of human T cells that secrete IFN-γ in response to the Melan A peptide, ELAGIGILTV, which corresponds to its HLA-A*0201-restricted human CD8+ T-cell epitope, *in vitro* (Figure 2B). Collectively, these data indicate that the NP vaccine triggers a potent Melan-A-specific CD8+ T cell response, both in term of frequency/number and cytokine production.

### The NP Vaccine Induces Expansion of Human *i*NKT Cells in HIS-CD8/NKT Mice

**FIGURE 3:**
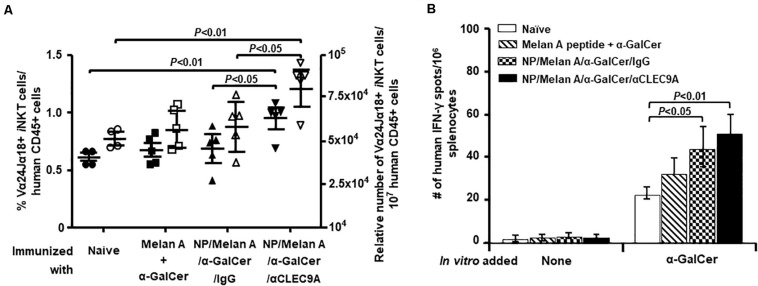
Level of α-GalCer-reactive human *i*NKT-cell response induced by NP vaccines. **(A)** The percentage (closed symbols) and number (open symbols) of Vα24^+^Jα18^+^ human *i*NKT cells among mouse CD45^+^ cells within splenocytes isolated from immunized HIS-CD8/NKT mice were determined by flow cytometry. **(B)** The relative number of α-GalCer-activated human *i*NKT cells that secrete IFN-γ among splenocytes from HIS-CD8/NKT mice immunized with NP-Melan-A vaccine was determined by an ELISpot assay. Results are displayed as the mean value and standard error (*n* = 5). Statistical analyses were performed using ANOVA followed by Dunnett’s test and differences were considered if *P* < 0.05.

We next determined whether α-GalCer encapsulated in the NP vaccine can activate and expand human *i*NKT cells in HIS-CD8/NKT mice. We stained PBMCs from all mice for an invariant TCR expressed by *i*NKT cells (Vα24/Jα18) and quantified the proportion of *i*NKT cells within all human CD45^+^ cells. As shown in Figure 3A, HIS-CD8/NKT mice that received NP/Melan A/α-GalCer/anti-CLEC9A had a significantly increased percentage and number of human *i*NKT cells compared to other control groups. Functional analysis of the *i*NKT cells by ELISpot to determine IFN-γ production in response to α-GalCer revealed a significantly higher number of IFN-γ-secreting *i*NKT cells not only in this animal group, but also in the group of mice that received the immunization with NP/Melan A/α-GalCer/IgG, compared to the group of mice immunized with Melan A peptide and α-GalCer (Figure 3B). The number of α-GalCer-reactive *i*NKT cells tended to be higher in the NP/Melan A/α-GalCer/anti-CLEC9A group compared to the control NP/Melan A/α-GalCer/IgG group, although the difference was not statistically significant (*p* = 0.135).

### The NP Vaccine Induces Expansion of CD141^+^ Human DCs in HIS-CD8/NKT Mice

**FIGURE 4:**
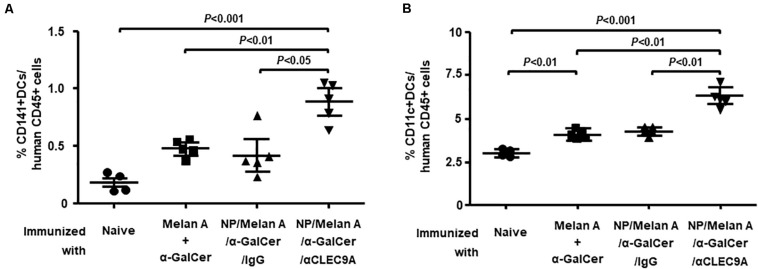
Expansion of human DC subsets by NP-Melan-A vaccines. **(A)** The percentage of CD141^+^ human DCs among mouse CD45^+^ cells within splenocytes isolated from immunized HIS-CD8/NKT mice was determined by flow cytometry. **(B)** The percentage of CD11c^+^ human DCs among CD45^+^ cells within splenocytes isolated from immunized HIS-CD8/NKT mice was determined by flow cytometry. Results are displayed as the mean value and standard error (*n* = 5). Statistical analyses were performed using ANOVA followed by Dunnett’s test and differences were considered if *P* < 0.05.

We next investigated the impact of the NP-based vaccine on the expansion of human CD141^+^ (BDCA3^+^) DCs. To this end, splenocytes collected from each of the vaccinated groups of HIS-CD8/NKT mice were incubated with anti-CD11c and anti-CD141 Abs then assessed by flow cytometry. As shown in Figure 4A, the percentage of CD141^+^ CD11c^+^ human DCs in the spleen of HIS-CD8/NKT mice that received NP/Melan A/α-GalCer/anti-CLEC9A significantly increased relative to the control. The percentage of CD11c^+^ DCs (pan-myeloid DCs) was also significantly increased in this mouse group (Figure 4B). The expansion of CD11c^+^ DCs that do not express CLEC9A, could be caused by their interaction with T cells and *i*NKT cells. Together, immunization with the NP vaccine decorated with the anti-CLEC9A Ab led to greater expansion of DCs, including CD141^+^ DCs, compared to HIS-CD8/NKT mice immunized with the control NP vaccine (IgG).

### The NP Vaccine Triggers Polyfunctional Human CD8^+^ T Cells as Revealed by Single Cell-Based Multiplexed Proteomics

**FIGURE 5:**
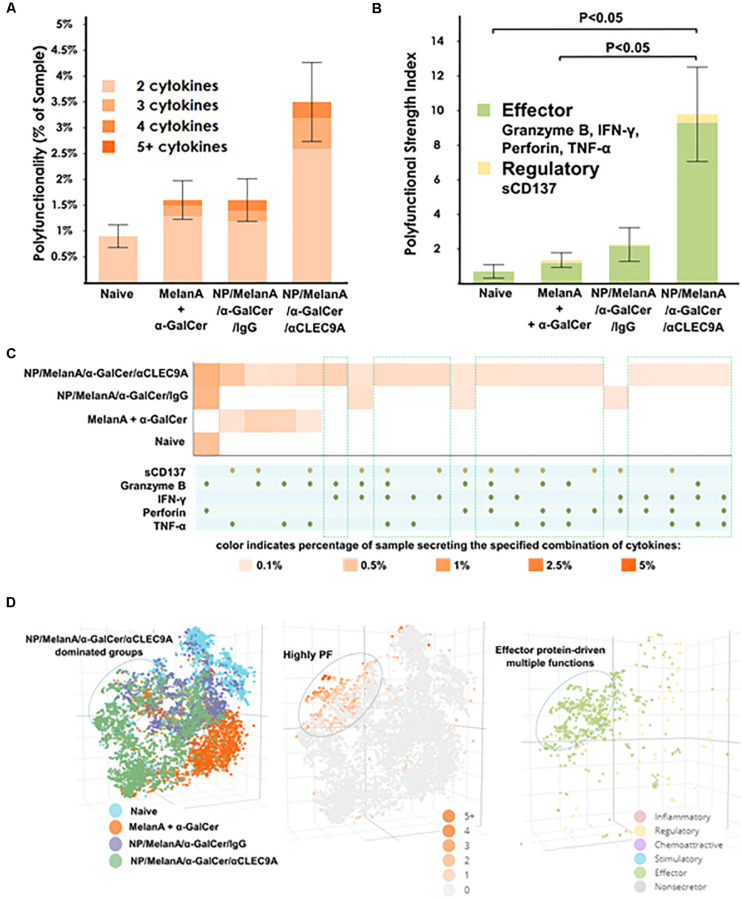
Polyfunctional human CD8^+^ T cells induced by the NP-Melan-A vaccines as determined by single cell-based multiplexed proteomics. **(A)** The NP-Melan-A vaccines resulted in an upregulation of polyfunctionality and **(B)** polyfunctional strength index (PSI). **(C)** Heat map identified an increase of polyfunctional cell subsets (multiple secreted cytokines) in the vaccinated mice compared to naive mice. Results are displayed as the mean value and standard error (*n* = 5). **(D)** 3D t-SNE visualization dissected high-dimensional single cells and revealed highly upregulated polyfunctionality of CD8^+^ T cells with effector protein-driven multiple functions upon NP-Melan-A vaccines. Statistical analyses were performed using Mann-Whitney *U* test and differences were considered if *P* < 0.05.

Lastly, we sought to evaluate the polyfunctional T-cell response in the immunized HIS-CD8/NKT mice. To this end, we took advantage of a 32-plex single-cell proteomics detection platform to profile human CD8^+^ T-cell functionality across approximately 500 single cells. The single-cell analysis identified a robust upregulation of polyfunctional human CD8^+^ T cells in the HIS-CD8/NKT mice immunized with NP/Melan-A/α-GalCer/anti-CLEC9A compared to other animal groups (Figure 5A). Polyfunctional T cells, defined as at least 2 proteins simultaneously co-secreted by a single cell, are recognized as one of the important functional attributes for the assessment of the T cell immune response to antigenic stimulation (30–32). As shown in Figure 5A, NP/Melan-A/α-GalCer/anti-CLEC9A elicited the highest increase of polyfunctional CD8^+^ T cells compared to NP/Melan-A/α-GalCer/anti-IgG and to Melan-A and α-GalCer administered in a free form. In addition, we noted a statistically significant difference between the NP/Melan A/α-GalCer/anti-CLEC9A group and both the mixture of soluble Melan A and α-GalCer and naïve groups when we calculated the polyfunctional strength index (PSI; see section “Materials and Methods”) of the human CD8^+^ T cells induced by the vaccines (Figure 5B). PSI was calculated based on the percentage of polyfunctional cells and the intensities of secreted proteins, suggesting the impact of NP/Melan-A/α-GalCer/anti-CLEC9A on promoting CD8^+^ T-cell polyfunctionality, as well as the quantity of each of the secreted proteins by these individual CD8^+^ T cells. Moreover, the significantly upregulated PSI of the human CD8^+^ T cells by the NP/Melan A/α-GalCer/anti-CLEC9A vaccine was predominated by anti-tumor associated proteins including human Granzyme B, IFN-γ, Perforin, and TNF-α, as well as a low level of sCD137 secretion (Figure 5B), indicating the superior anti-cancer potential of this vaccine.

Visualization of each of the combination of proteins as a heatmap revealed specific subsets of polyfunctional cells upregulated by the NP/Melan A/α-GalCer/anti-CLEC9A vaccine (Figure 5C). For example, polyfunctional CD8^+^ T cells which expressed both Granzyme B and Perforin, or those that expressed TNF-α and sCD137 together, were upregulated in the NP/Melan A/α-GalCer/anti-CLEC9A group compared to other groups. Breaking down the contribution of each protein to the overall PSI indicated that all the identified proteins were major contributors to the PSI observed (Supplementary Figure S3). In addition, as shown in Figure 5D, non-linear single-cell visualization by three-dimensional t-distributed stochastic neighbor embedding (3D t-SNE), a machine-learning algorithm (35), revealed that cells isolated from mice which received the NP/Melan A/α-GalCer/anti-CLEC9A vaccine clustered separately from cells of other mice, and that this group was highly polyfunctional and exhibited a profile of multiple effector-driven functions.

## Discussion

Growing evidence demonstrates that *i*NKT cell agonists (typically α-GalCer) may directly potentiate anti-cancer therapy. However, innovative strategies to better manipulate the adjuvant effects against *i*NKT cells are required. In the current study, we tested the ability of an NP-based vaccine to enhance DC/*i*NKT cell/naïve CD8^+^ T cell interactions, thereby triggering potent Ag-specific CD8^+^ T responses. This design was based on exploiting the unique ability of human *i*NKT cells to substitute “classical” CD4^+^ T helper cells in licensing cross-priming DCs *in vivo* (36). We have recently demonstrated the ability of a nanovaccine containing the Melan A Ag and α-GalCer and coated with anti-CLEC9A Abs to strongly induce the expansion of tumor Ag-specific CD8^+^ T cells from human PBMCs *in vitro* (12). In order to determine whether such an NP-based Melan A vaccine can successfully induce a tumor Ag-specific human CD8^+^ T cell response “*in vivo*,” we have utilized a humanized mouse model, which mimics the human immune system and includes functional human CD8^+^ T cells (28), *i*NKT cells (27), and human CD141^+^ DCs (29). These humanized mice, called HIS-CD8/NKT mice, are made by transducing β2-microglobulin-deficient NSG mice (lack mouse CD1d) with HLA-A2/human β2m and human CD1d/human β2m, as well as human hematopoietic cytokines (human GM-CSF, IL-3, and IL-15), followed by the engraftment of A2-matched human hematopoietic stem cells (27–29).

Our main finding is that the NP-based Melan A vaccine was able to induce a high level of Melan-A-specific, A2-restricted human CD8^+^ T-cell response in HIS-CD8/NKT mice (Figure 2). Furthermore, the response to the vaccine included a significant level of human *i*NKT cell (Figure 3) and human CD141^+^ DCs (Figure 4A) responses. We further profiled the functionality of the human CD8^+^ T cells induced by the NP vaccine by using a 32-plex single-cell proteomics detection platform, finding that there was a robust upregulation of polyfunctionality due to the vaccine (Figure 5). We found that these polyfunctional CD8^+^ T cells secreted greater levels of granzyme B, IFN-γ, perforin, TNF-α, and sCD137 in the NP vaccine-immunized HIS-CD8/NKT mice relative to other groups. We employed 3D t-SNE, a non-linear dimensionality reduction tool to dissect the high-dimensional single-cell data and demonstrate distinct cell clusters among the groups with a prominent increase of polyfunctionality in CD8^+^ T cells by NP vaccine. Furthermore, these polyfunctional cell subsets promoted by NP/Melan A/α-GalCer/anti-CLEC9A vaccine predominantly secreted effector proteins that are associated with antitumor immunity. Hence, the use of this humanized mouse model allowed us to accurately replicate the human *i*NKT cell response and assess a novel vaccination strategy.

Two independent studies have recently used α-GalCer or its analog as a means to enhance a tumor-specific CD8^+^ T-cell response (24, 25). In the first study, which was conducted by our group, mice were immunized with tumor peptides simply mixed with a free form of an α-GalCer analog, and we observed a slight increase in tumor-specific CD8^+^ T-cell response and protective immunity, both of which were further enhanced by co-administration of monophosphoryl lipid A (MPLA), a TLR4 agonist (24). Our study indicated that the α-GalCer analog can induce robust CD8^+^ T-cell-mediated protective immunity when co-administered with MPLA, thus rendering this combination of adjuvants a novel vaccine adjuvant formulation. The second study by Dölen, Y. et al. compared the immunogenicity and efficacy of a PGLA-based NP vaccine that encapsulated both a full-length ovalbumin (OVA) and α-GalCer with those of PGLA that encapsulated OVA and a several TLR ligands (R848 and poly I:C) (25). They compared the level of OVA-specific T-cell responses, as well as the protective efficacy of these responses by challenging mice with a B16 melanoma cell line that expressed OVA. The study concluded that the vaccine which co-encapsulated OVA and α-GalCer exerted a more robust T-cell-mediated anti-tumor response than the vaccine which co-encapsulated OVA and a TLR ligand. This current study is in agreement with these two previous studies in demonstrating α-GalCer is a potent adjuvant for a tumor-specific T-cell response. However, in contrast to both published studies, which were done using conventional mice, this current work has demonstrated in a human immune system model that a PGLA-based NP vaccine which encapsulated a tumor antigen and α-GalCer and decorated by anti-CLEC9A can actually engage both populations of human iNKT cells and human CD141^+^ DCs *in vivo*. In doing so, the vaccine successfully expanded DC/*i*NKT cell/naïve CD8^+^ T cell interactions, thereby efficiently priming and expanding Ag-specific CD8^+^ T cells as intended, and ultimately enhanced a tumor Ag-specific human CD8^+^ T-cell response. With regards to the anti-tumor effects of the tumor Ag-specific human CD8^+^ T cells both *in vivo* and *in vitro*, we are planning to determine whether NP formulation is able to induce such tumoricidal human CD8^+^ T cells in HIS-CD8/NKT mice using an appropriate tumor model in the near future.

In summary, we demonstrated the successful induction of a significant level of tumor Ag-specific human CD8^+^ T-cell response in HIS-CD8/NKT mice by an NP-based vaccine. As one of the advantages of using such a humanized mouse model was that we could visibly monitor the interactions of important cell types including human CD8^+^ T cells, *i*NKT cells, and CD141^+^ DCs, future studies could address mechanistic properties through methods such as an intravital imaging analysis by multi-photon microscopy (37, 38) and a whole body *in vivo* imaging system (39, 40). Secondly and more importantly, we will be able to challenge NP vaccine-immunized HIS-CD8/NKT mice with Melan A-expressing tumors and determine the efficacy of the vaccine. Finally, the successful profiling of the NP vaccine-induced polyfunctional human CD8^+^ T cells using single-cell proteomics may serve as predictive biomarkers for a more accurate evaluation of tumor vaccine efficacy in a pre-clinical setting.

## Data Availability Statement

All datasets presented in this study are included in the article/Supplementary Material.

## Ethics Statement

All animal experiments were conducted in strict accordance with the Policy on Humane Care and Use of Laboratory Animals of the United States Public Health Service. The protocol was approved by the Institutional Animal Care and Use Committee at the Rockefeller University (Assurance # A3081-01).

## Author Contributions

JZ, PP, and SM contributed to the design and execution of single-cell experiments, their data analyses and interpretation, and writing of the manuscript related to the single-cell experiments. LC prepared the NP vaccine. RG and DS validated the immunogenicity and the specificity of the NP vaccine (human setting). MT and FT designed the *in vivo* experiments. JH, YT, and MT generated humanized mice. JH, TS, and MT conducted the *in vivo* experiments. SI, FT, and MT analyzed the *in vivo* data and wrote the manuscript. All authors contributed to the article and approved the submitted version.

## Conflict of Interest

JZ is employed by and has equity ownership in IsoPlexis. PP is employed by, has equity ownership in, and is a patent holder with IsoPlexis. SM is cofounder of, has equity ownership in, and is a patent holder with IsoPlexis. The remaining authors declare that the research was conducted in the absence of any commercial or financial relationships that could be construed as a potential conflict of interest.

## Supplementary Material

FIGURE S1 Lack of cross-reactivity of the human DC-targeting Ab used in this study, anti-CLEC9A, to mouse DCs. Anti-Clec9a Ab, the murine equivalent, reacts with mouse DCs and is shown as a control.

FIGURE S2 Representative flow cytometry profiles of splenocytes isolated from NP-Melan A vaccine-immunized HIS-CD8/NKT mice by the Melan-A tetramer.

FIGURE S3 Breakdown of the contribution of individual proteins to the overall PSI. All proteins, Granzyme B, IFN-γ, Perforin, TNF-α, and sCD137, contributed to the significantly upregulated PSI induced by the NP/Melan-A/αGalCer/anti-CLEC9A vaccine.

## References

[1] Klebanoff CA Acquavella N Yu Z Restifo NP. Therapeutic cancer vaccines: are we there yet? *Immunol Rev.* (2011) 239:27–44. 10.1111/j.1600-065X.2010.00979.x 21198663 PMC3075547

[2] Chen DS Mellman I. Oncology meets immunology: the cancer-immunity cycle. *Immunity.* (2013) 39:1–10. 10.1016/j.immuni.2013.07.012 23890059

[3] Palucka K Banchereau J. Dendritic-cell-based therapeutic cancer vaccines. *Immunity.* (2013) 39:38–48. 10.1016/j.immuni.2013.07.004 23890062 PMC3788678

[4] Qiu L Valente M Dolen Y Jäger E Beest MT Zheng L Endolysosomal-escape nanovaccines through adjuvant-induced tumor antigen assembly for enhanced effector CD8(+) T cell activation. *Small.* (2018) 14:e1703539. 10.1002/smll.201703539 29493121

[5] Amador-Molina A Trejo-Moreno C Romero-Rodríguez D Sada-Ovalle I Pérez-Cárdenas E Lamoyi E Vaccination with human papillomavirus-18 E1 protein plus α-galactosyl-ceramide induces CD8(+) cytotoxic response and impairs the growth of E1-expressing tumors. *Vaccine.* (2019) 37:1219–28. 10.1016/j.vaccine.2018.12.036 30704821

[6] Moyer TJ Zmolek AC Irvine DJ. Beyond antigens and adjuvants: formulating future vaccines. *J Clin Invest.* (2016) 126:799–808. 10.1172/JCI81083 26928033 PMC4767337

[7] Bobbala S Hook S. Is there an optimal formulation and delivery strategy for subunit vaccines? *Pharm Res.* (2016) 33:2078–97. 10.1007/s11095-016-1979-0 27380191

[8] Shah RR Hassett KJ Brito LA. Overview of vaccine adjuvants: introduction, history, and current status. *Methods Mol Biol.* (2017) 1494:1–13. 10.1007/978-1-4939-6445-1_1 27718182

[9] O’Hagan DT Fox CB. New generation adjuvants–from empiricism to rational design. *Vaccine.* (2015) 33(Suppl. 2):B14–20. 10.1016/j.vaccine.2015.01.088 26022561

[10] Silva AL Soema PC Slutter B Ossendorp F Jiskoot W. PLGA particulate delivery systems for subunit vaccines: Linking particle properties to immunogenicity. *Hum Vaccin Immunother.* (2016) 12:1056–69. 10.1080/21645515.2015.1117714 26752261 PMC4962933

[11] Koerner J Horvath D Groettrup M. Harnessing dendritic CElls for poly (D,L-lactide-co-glycolide) microspheres (PLGA MS)–mediated anti-tumor therapy. *Front Immunol.* (2019) 10:707. 10.3389/fimmu.2019.00707 31024545 PMC6460768

[12] Ghinnagow R De Meester J Cruz LJ Aspord C Corgnac S Macho-Fernandez E Co-delivery of the NKT agonist alpha-galactosylceramide and tumor antigens to cross-priming dendritic cells breaks tolerance to self-antigens and promotes antitumor responses. *Oncoimmunology.* (2017) 6:e1339855. 10.1080/2162402X.2017.1339855 28932640 PMC5599097

[13] Sancho D Mourão-Sá D Joffre OP Schulz O Rogers NC Pennington DJ Tumor therapy in mice via antigen targeting to a novel, DC-restricted C-type lectin. *J Clin Invest.* (2008) 118:2098–110. 10.1172/JCI34584 18497879 PMC2391066

[14] Paulis LE Mandal S Kreutz M Figdor CG. Dendritic cell-based nanovaccines for cancer immunotherapy. *Curr Opin Immunol.* (2013) 25:389–95. 10.1016/j.coi.2013.03.001 23571027

[15] Macho-Fernandez E Cruz LJ Ghinnagow R Fontaine J Bialecki E Frisch B Targeted delivery of alpha-galactosylceramide to CD8alpha+ dendritic cells optimizes type I NKT cell-based antitumor responses. *J Immunol.* (2014) 193:961–9. 10.4049/jimmunol.1303029 24913977

[16] Picco G Beatson R Taylor-Papadimitriou J Burchell JM. Targeting DNGR-1 (CLEC9A) with antibody/MUC1 peptide conjugates as a vaccine for carcinomas. *Eur J Immunol.* (2014) 44:1947–55. 10.1002/eji.201344076 24648154 PMC4209794

[17] Dhodapkar MV Sznol M Zhao B Wang D Carvajal RD Keohan ML Induction of antigen-specific immunity with a vaccine targeting NY-ESO-1 to the dendritic cell receptor DEC-205. *Sci Transl Med.* (2014) 6:232ra251. 10.1126/scitranslmed.3008068 24739759 PMC6151129

[18] Silva JM Videira M Gaspar R Preat V Florindo HF. Immune system targeting by biodegradable nanoparticles for cancer vaccines. *J Control Release.* (2013) 168:179–99. 10.1016/j.jconrel.2013.03.010 23524187

[19] Temizoz B Kuroda E Ishii KJ. Vaccine adjuvants as potential cancer immunotherapeutics. *Int Immunol.* (2016) 28:329–38. 10.1093/intimm/dxw015 27006304 PMC4922024

[20] Gonzalez-Aseguinolaza G Van Kaer L Bergmann CC Wilson JM Schmieg J Kronenberg M Natural killer T cell ligand alpha-galactosylceramide enhances protective immunity induced by malaria vaccines. *J Exp Med.* (2002) 195:617–24. 10.1084/jem.20011889 11877484 PMC2193764

[21] Hermans IF Silk JD Gileadi U Salio M Mathew B Ritter G NKT cells enhance CD4+ and CD8+ T cell responses to soluble antigen in vivo through direct interaction with dendritic cells. *J Immunol.* (2003) 171:5140–7. 10.4049/jimmunol.171.10.5140 14607913

[22] Fujii S Shimizu K Smith C Bonifaz L Steinman RM. Activation of natural killer T cells by alpha-galactosylceramide rapidly induces the full maturation of dendritic cells in vivo and thereby acts as an adjuvant for combined CD4 and CD8 T cell immunity to a coadministered protein. *J Exp Med.* (2003) 198:267–79. 10.1084/jem.20030324 12874260 PMC2194082

[23] Stober D Jomantaite I Schirmbeck R Reimann J. NKT cells provide help for dendritic cell-dependent priming of MHC class I-restricted CD8+ T cells in vivo. *J Immunol.* (2003) 170:2540–8. 10.4049/jimmunol.170.5.2540 12594280

[24] Coelho-Dos-Reis JG Huang J Tsao T Pereira FV Funakoshi R Nakajima H Co-administration of α-GalCer analog and TLR4 agonist induces robust CD8(+) T-cell responses to PyCS protein and WT-1 antigen and activates memory-like effector NKT cells. *Clin Immunol.* (2016) 168:6–15. 10.1016/j.clim.2016.04.014 27132023 PMC4940295

[25] Dölen Y Kreutz M Gileadi U Tel J Vasaturo A van Dinther EA Co-delivery of PLGA encapsulated invariant NKT cell agonist with antigenic protein induce strong T cell-mediated antitumor immune responses. *Oncoimmunology.* (2015) 5:e1068493. 10.1080/2162402X.2015.1068493 26942088 PMC4760331

[26] Tullett KM Lahoud MH Radford KJ. Harnessing human cross-presenting CLEC9A(+)XCR1(+) dendritic cells for immunotherapy. *Front Immunol.* (2014) 5:239. 10.3389/fimmu.2014.00239 24904587 PMC4033245

[27] Li X Huang J Kaneko I Zhang M Iwanaga S Yuda M A potent adjuvant effect of a CD1d-binding NKT cell ligand in human immune system mice. *Expert Rev Vaccines.* (2017) 16:73–80. 10.1080/14760584.2017.1256208 27801602 PMC5526659

[28] Huang J Li X Coelho-dos-Reis JG Wilson JM Tsuji M. An AAV vector-mediated gene delivery approach facilitates reconstitution of functional human CD8+ T cells in mice. *PLoS One.* (2014) 9:e88205. 10.1371/journal.pone.0088205 24516613 PMC3916402

[29] Coelho-Dos-Reis JGA Funakoshi R Huang J Pereira FV Iketani S Tsuji M. Functional human CD141+ dendritic cells in human immune system mice. *J Infect Dis.* (2020) 221:201–13. 10.1093/infdis/jiz432 31647546 PMC7457331

[30] Zhou J Kaiser A Ng C Karcher R McConnell T Paczkowski P CD8+ T-cell mediated anti-malaria protection induced by malaria vaccines; assessment of hepatic CD8+ T cells by SCBC assay. *Hum Vaccin Immunother.* (2017) 13:1625–9. 10.1080/21645515.2017.1304333 28362549 PMC5512776

[31] Xue Q Bettini E Paczkowski P Ng C Kaiser A McConnell T Single-cell multiplexed cytokine profiling of CD19 CAR-T cells reveals a diverse landscape of polyfunctional antigen-specific response. *J Immunother Cancer.* (2017) 5:85. 10.1186/s40425-017-0293-7 29157295 PMC5697351

[32] Rossi J Paczkowski P Shen YW Morse K Flynn B Kaiser A Preinfusion polyfunctional anti-CD19 chimeric antigen receptor T cells are associated with clinical outcomes in NHL. *Blood.* (2018) 132:804–14. 10.1182/blood-2018-01-828343 29895668 PMC6107882

[33] Parisi G Saco JD Salazar FB Tsoi J Krystofinski P Saus CP Persistence of adoptively transferred T cells with a kinetically engineered IL-2 receptor agonist. *Nat Commun.* (2020) 11:660. 10.1038/s41467-019-12901-3 32005809 PMC6994533

[34] Ma C Cheung AF Chodon T Koya RC Wu Z Ng C Multifunctional T-cell analyses to study response and progression in adoptive cell transfer immunotherapy. *Cancer Discov.* (2013) 3:418–29. 10.1158/2159-8290.CD-12-0383 23519018 PMC3716460

[35] Kobak D Berens P. The art of using t-SNE for single-cell transcriptomics. *Nat Commun.* (2019) 10:5416. 10.1038/s41467-019-13056-x 31780648 PMC6882829

[36] Semmling V Lukacs-Kornek V Thaiss CA Quast T Hochheiser K Panzer U Alternative cross-priming through CCL17-CCR4-mediated attraction of CTLs toward NKT cell-licensed DCs. *Nat Immunol.* (2010) 11:313–20. 10.1038/ni.1848 20190758

[37] van Panhuys N. Studying dendritic Cell-T cell interactions under in vivo conditions. *Methods Mol Biol.* (2017) 1584:569–83. 10.1007/978-1-4939-6881-7_36 28255727

[38] Wang L Xie Y Ahmed KA Ahmed S Sami A Chibbar R Exosomal pMHC-I complex targets T cell-based vaccine to directly stimulate CTL responses leading to antitumor immunity in transgenic FVBneuN and HLA-A2/HER2 mice and eradicating trastuzumab-resistant tumor in athymic nude mice. *Breast Cancer Res Treat.* (2013) 140:273–84. 10.1007/s10549-013-2626-7 23881522

[39] Ottobrini L Martelli C Trabattoni DL Clerici M Lucignani G. In vivo imaging of immune cell trafficking in cancer. *Eur J Nucl Med Mol Imaging.* (2011) 38:949–68. 10.1007/s00259-010-1687-7 21170525

[40] Prins RM Shu CJ Radu CG Vo DD Khan-Farooqi H Soto H Anti-tumor activity and trafficking of self, tumor-specific T cells against tumors located in the brain. *Cancer Immunol Immunother.* (2008) 57:1279–89. 10.1007/s00262-008-0461-1 18253732 PMC2614264

