# Supplementary figures and images for: Targeted Co-delivery of Tumor Antigen and α-Galactosylceramide to CD141+ Dendritic Cells Induces a Potent Tumor Antigen-Specific Human CD8+ T Cell Response in Human Immune System Mice
Source: Front Immunol. 2020 Aug 18;11:2043. doi: 10.3389/fimmu.2020.02043 (PMC7461784; doi:10.3389/fimmu.2020.02043)

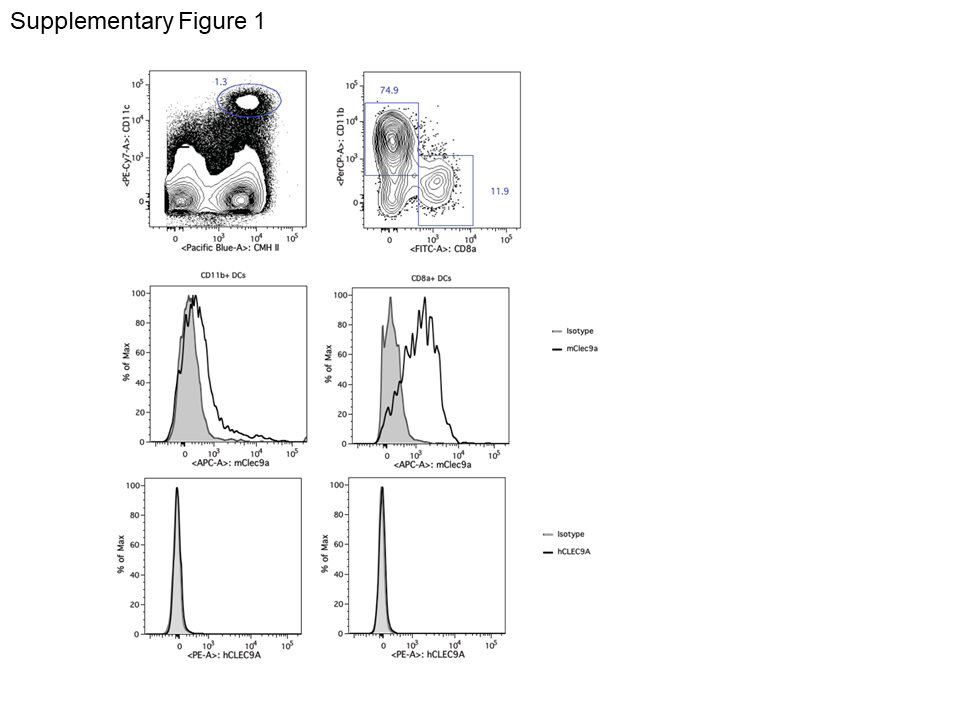

Supplement: FIGURE S1 — Lack of cross-reactivity of the human DC-targeting Ab used in this study, anti-CLEC9A, to mouse DCs. Anti-Clec9a Ab, the murine equivalent, reacts with mouse DCs and is shown as a control. [file Image_1.TIF]

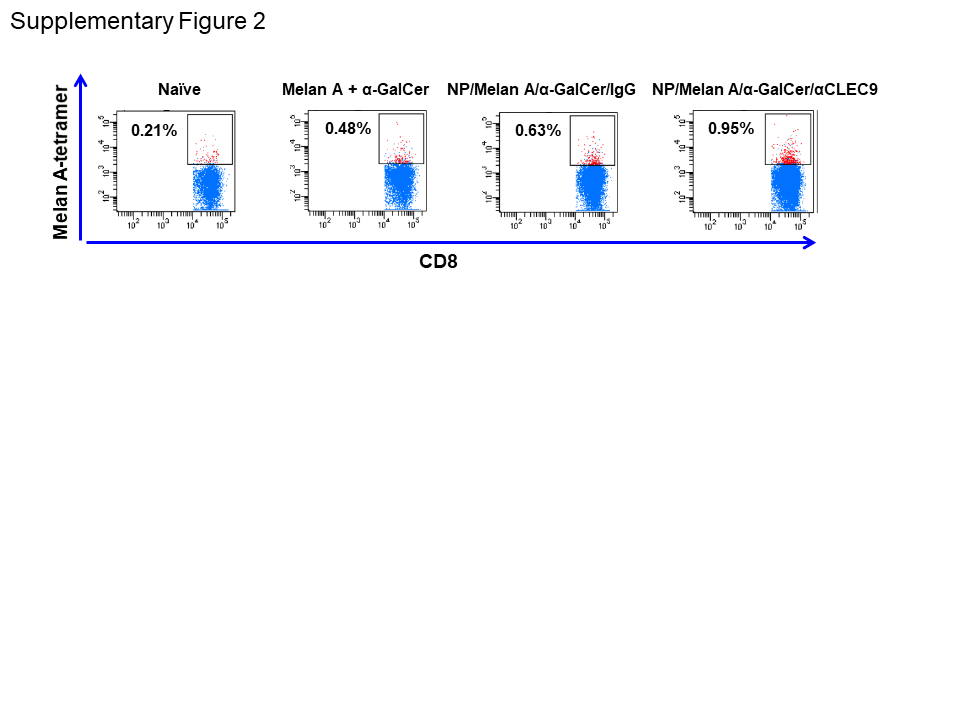

Supplement: FIGURE S2 — Representative flow cytometry profiles of splenocytes isolated from NP-Melan A vaccine-immunized HIS-CD8/NKT mice by the Melan-A tetramer. [file Image_2.TIF]

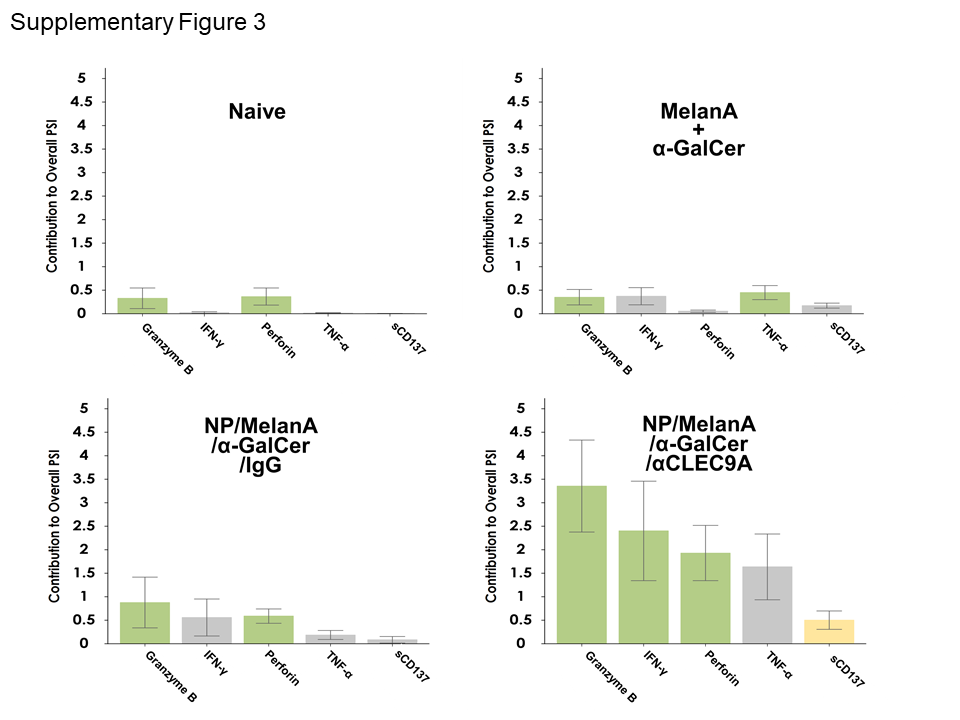

Supplement: FIGURE S3 — Breakdown of the contribution of individual proteins to the overall PSI. All proteins, Granzyme B, IFN-γ, Perforin, TNF-α, and sCD137, contributed to the significantly upregulated PSI induced by the NP/Melan-A/αGalCer/anti-CLEC9A vaccine. [file Image_3.TIF]
